# A Logic Model of Neuronal-Glial Interaction Suggests Altered Homeostatic Regulation in the Perpetuation of Neuroinflammation

**DOI:** 10.3389/fncel.2018.00336

**Published:** 2018-10-15

**Authors:** Travis J. A. Craddock, Lindsay T. Michalovicz, Kimberly A. Kelly, Mark A. Rice, Diane B. Miller, Nancy G. Klimas, Mariana Morris, James P. O'Callaghan, Gordon Broderick

**Affiliations:** ^1^Department of Psychology & Neuroscience, Nova Southeastern University, Ft. Lauderdale, FL, United States; ^2^Institute for Neuro-Immune Medicine, Nova Southeastern University, Ft. Lauderdale, FL, United States; ^3^Department of Computer Science, Nova Southeastern University, Ft. Lauderdale, FL, United States; ^4^Department of Clinical Immunology, Nova Southeastern University, Ft. Lauderdale, FL, United States; ^5^Health Effects Laboratory Division, Centers for Disease Control and Prevention, National Institute for Occupational Safety and Health, Morgantown, WV, United States; ^6^Miami Veterans Affairs Medical Center, Miami, FL, United States; ^7^Center for Clinical Systems Biology, Rochester General Hospital, Rochester, NY, United States

**Keywords:** neuroinflammation, logical modeling, systems neuroscience, regulatory biology, homeostatic regulation, treatment course prediction, neural glial interaction, mouse models

## Abstract

Aberrant inflammatory signaling between neuronal and glial cells can develop into a persistent sickness behavior-related disorders, negatively impacting learning, memory, and neurogenesis. While there is an abundance of literature describing these interactions, there still lacks a comprehensive mathematical model describing the complex feed-forward and feedback mechanisms of neural-glial interaction. Here we compile molecular and cellular signaling information from various studies and reviews in the literature to create a logically-consistent, theoretical model of neural-glial interaction in the brain to explore the role of neuron-glia homeostatic regulation in the perpetuation of neuroinflammation. Logic rules are applied to this connectivity diagram to predict the system's homeostatic behavior. We validate our model predicted homeostatic profiles against RNAseq gene expression profiles in a mouse model of stress primed neuroinflammation. A meta-analysis was used to calculate the significance of similarity between the inflammatory profiles of mice exposed to diisopropyl fluorophostphate (DFP) [with and without prior priming by the glucocorticoid stress hormone corticosterone (CORT)], with the equilibrium states predicted by the model, and to provide estimates of the degree of the neuroinflammatory response. Beyond normal homeostatic regulation, our model predicts an alternate self-perpetuating condition consistent with chronic neuroinflammation. RNAseq gene expression profiles from the cortex of mice exposed to DFP and CORT+DFP align with this predicted state of neuroinflammation, whereas the alignment to CORT alone was negligible. Simulations of putative treatment strategies post-exposure were shown to be theoretically capable of returning the system to a state of typically healthy regulation with broad-acting anti-inflammatory agents showing the highest probability of success. The results support a role for the brain's own homeostatic drive in perpetuating the chronic neuroinflammation associated with exposure to the organophosphate DFP, with and without CORT priming. The deviation of illness profiles from exact model predictions suggests the presence of additional factors or of lasting changes to the brain's regulatory circuitry specific to each exposure.

## Introduction

Neuroinflammation, the enhanced expression of inflammatory mediators in the brain, has been associated with a variety of central nervous system (CNS) disorders, including cognitive dysfunction, mental illness, and neurodegeneration. Neuroinflammation can result from a variety of factors including infection, traumatic brain injury, chemical exposures, toxic metabolites, or autoimmune insults, both centrally and peripherally. The complex cross-talk among neuronal and glial cells of the CNS, comprised of multiple feed-forward and feed-back mechanisms, is essential to maintaining proper homeostasis within the brain and supporting appropriate neuroinflammatory responsiveness to physical, psychological, or immune stressors (reviewed in (Kettenmann et al., 2011; Kierdorf and Prinz, 2013; Xanthos and Sandkühler, 2014). The effects of the neuroinflammatory response are highly complex, with evidence of both neuroprotective and neurotoxic actions of activated glial cells. A greater understanding of such disparate signaling could facilitate targeted therapeutic strategies that aim to diminish the adverse effects of neuroinflammation, while maintaining the benefits (Sofroniew and Vinters, 2010).

Neuroinflammation can be a response to peripheral inflammatory insults or arise from a direct insult to the CNS itself. Numerous studies have shown the body's ability to communicate inflammatory information to the brain via both humoral and neuronal mechanisms (Hermans et al., 2011; Jurgens and Johnson, 2012), resulting in behavioral changes deemed “sickness behavior” (Dantzer et al., 2008; Koo and Duman, 2008). Processing of this inflammatory information by the brain can result in cellular activation and the production of inflammatory cytokines within the CNS. Additionally, exposure to severe or chronic psychological stressors can result in the activation of CNS resident cells, particularly microglia, and in the production of high levels of cytokines (Hinwood et al., 2012; Iwata et al., 2013; Calcia et al., 2016). Studies have shown that glial cells are responsive to many stimuli, expressing different transcription profiles depending on the initial triggering factor (Ekdahl, 2012; Zamanian et al., 2012). Immune responses in the context of an ongoing exposure to repeated stressors or a stressor subsequent to an underlying immune reaction can potentiate such cytokine responses further perpetuating neuroinflammation (Frank et al., 2016).

Aside from their inflammatory role, cytokines are intimately involved in behavioral modification, contributing to both molecular and cellular mechanisms that underlie learning, memory and cognition (McAfoose and Baune, 2009). Indeed, disruption of cellular communication due to inflammatory insult to the brain can result in unregulated neurotransmitter and hormone expression, as well as elevated pro-inflammatory cytokine expression with the latter being known to negatively impacting learning, and memory (Jurgens and Johnson, 2012; Kierdorf and Prinz, 2013; Kohman and Rhodes, 2013) in conditions such as major depression, dementia and Alzheimer's disease (Licastro et al., 2000; Cacquevel et al., 2004; McAfoose and Baune, 2009). It has been hypothesized that such elevated levels of proinflammatory cytokines, in the absence of compensatory mechanisms to regulate their effects, can reduce normal neuronal function such that microglia escape regulatory control, become excessively activated and promote further inflammation in a self-sustaining cascade (Yirmiya and Goshen, 2011; Jurgens and Johnson, 2012). This suggests a role for altered neuronal-glial homeostasis in the perpetuation of chronic neuroinflammation.

Clearly, neuroimmune signaling is highly complex, with communication between neuronal and glial cells being regulated by several neurotransmitters, hormones and cytokines with the potential for combinatorial signaling cascades (Sofroniew and Vinters, 2010; Tian et al., 2012). While there is an abundance of literature describing signaling interactions between cell types, these are often presented as linear in nature, devoid of important feed-forward and feedback mechanisms. Moreover, rarely are these models described with mathematical rigor. Such context-dependent combinatorial signaling involving multiple cell types can potentially stimulate cells to produce cytotoxic levels of signaling molecules, which may in turn support chronic neuroinflammation. Given the potentially important role of chronic neuroinflammation in the development of cognitive dysfunction and mental illness, we conducted a broad survey of molecular and cellular signaling mechanisms reported in various studies and reviews to create a comprehensive and consistent, theoretical model of neuronal-glial interaction logic. Such a model can be used to integrate the regulation of microglia with other immune mechanisms in the brain while also incorporating the complex signaling linking the many cell types involved (Craddock et al., 2014; Fritsch et al., 2014). Ultimately, knowledge of such homeostatic regulation can be used to identify promising applications of pharmaceutical, hormone and/or immune therapy that exploit the body's natural regulation (Craddock et al., 2015) to reinforce treatment effects in illnesses characterized by chronic neuroinflammation, such as Alzheimer's disease, Parkinson's disease, major depression, Myalgic Encephalomyelitis/Chronic Fatigue Syndrome (ME/CFS) and Gulf War Illness (GWI).

Based on our previous work (Craddock et al., 2014; Fritsch et al., 2014), we hypothesize that the complexity of the neuroimmune signaling system can allow for multiple regulatory modes beyond what is typically considered typical health. We validated this model by comparing its predicted stable states with gene expression profiles in the cortex of mice exposed to the organophosphate, sarin surrogate, diisopropyl fluorophostphate (DFP), both in the presence and absence of priming with the anti-inflammatory glucocorticoid and stress hormone, corticosterone (CORT), as this has previously been shown to exacerbate the neuroinflammatory response (O'Callaghan et al., 2015; Locker et al., 2017). We find that the literature-based logic model accommodates normal regulation of neuroimmunity as well as an alternate regulatory mode indicative of chronic neuroinflammation. The marker expression profile predicted at this alternate steady state shows agreement with the neuroinflammatory profile presented in the mouse model by exposure to DFP in both the absence and presence of CORT priming. Simulations of putative treatment strategies targeting microglia, or the immune and glucocorticoid systems, were shown to be theoretically capable of returning the system to a state of typically healthy regulation.

## Materials and methods

### A discrete integrative model of the neuro-immune system

There is a substantial body of literature describing the innate immunological responses of the nervous system, involving microglia, astrocytes, cytokines, chemokines, and related molecular processes. As much of the experimental data describing neuroimmune processes currently requires invasive sampling, it is necessary to use animal models or post-mortem human brain tissue to obtain estimates of the stoichiometry and kinetics of interactions between cells and molecules. However, even in these situations, the response rates and associated parameters can be highly context dependent. To circumvent this obstacle, we used a discrete logic modeling methodology to provide a qualitative description of system behavior that is based primarily on cause-and-effect connectivity and is independent of specific kinetic parameters. As opposed to conventional formalisms where the abundance of cells and the concentration of molecular markers are expressed along a continuum of values, entities in the discrete model do not vary smoothly, but rather have distinct, separate expression levels (e.g., high, normal, low expressed as integer numbers: 0, 1, 2 … etc.).

Here we used mechanistic interpretations published in the literature to infer the stimulatory and inhibitory interactions linking the various cellular and molecular elements of the model neuroimmune network (i.e., the network topology). Details describing each interaction are presented in the section entitled “Results: Literature Based Discrete Neuro-Immune Signaling Network.” The evolution in time of the system was simulated using a discrete event formalism based on seminal work by Kauffman (1969); Thomas (1991) and Mendoza and Xenarios (2006), that was subsequently extended by our group (Craddock et al., 2014). At its simplest, feedback and feedforward control dynamics are represented solely by the direction (source and target) and type (stimulator or inhibitor) of interaction and the execution of any given actions was scheduled asynchronously in an attempt to better represent biological variability. In this first approximation, we assume cell types and molecular species to have the same regulatory signal strength on their respective targets and express with relatively uniform concentrations across small anatomical regions of the brain, as data regarding broad spatial distribution of these is limited at this time. Similarly, the corresponding molecular or cellular response to signaling is considered uniform in range.

### Discrete 3-valued logical analysis

The direction and type of interaction is sufficient to support the mathematical modeling of the stimulatory and inhibitory control patterns that determine the state of the overall signaling network (for a review see De Jong, 2002). As mentioned above, using the discrete formalism developed in Craddock et al. (2014), we can determine the number and qualitative signature of each stable state available to the model neuroimmune regulatory system without requiring detailed kinetic information. That is, we can determine where the system will eventually come to rest even though we may not know how quickly this state will be reached. In this model, signaling molecules and cell types are represented as individual variables, each capable of adopting 3 discrete states: −1 (below typical health), 0 (typical health), and 1 (above typical health). At any point in time t, the state of a system with N variables can be represented by the vector $\vec{x}\left(t\right)$, such that:

(1) $$\vec{x}\left(t\right)=\left(x_{1}\left(t\right),x_{2}\left(t\right),\ldots ,x_{N}\left(t\right)\right)$$

where *x*_*i*_(*t*) is the state of the i^th^ variable of the N variable system at time t. The image vector $\vec{x}\left(t+1\right)$ describes the preferred state toward which the system evolves in the next time increment. The state value of the image vector for the i^th^ variable is determined from its current state and a set of balanced ternary logic statements based on the current value of the variable and the mode of action (i.e., stimulate or inhibit) of the neighboring input variables. These logic statements are expressed as follows (Equation 2):

(2) $$\begin{array}{l} x_{i}\left(t+1\right)\\ =\{\begin{array}{c} \left(x_{i1}^{A}\left(t\right)\vee x_{i2}^{A}\left(t\right)\ldots x_{ij}^{A}\left(t\right))\nabla (x_{i1}^{I}\left(t\right)\vee x_{i2}^{I}\left(t\right)\ldots x_{ik}^{I}\left(t\right)\right)\\ \left(x_{i1}^{A}\left(t\right)\vee x_{i2}^{A}\left(t\right)\ldots x_{ij}^{A}\left(t\right)\right)\\ \neg (x_{i1}^{I}\left(t\right)\vee x_{i2}^{I}\left(t\right)\ldots x_{ik}^{I}\left(t\right)) \end{array} \end{array}$$

where the ∇, ∨, and ¬ symbols are ternary HIGH/LOW PASS, OR, and NOT operators, $x_{ij}^{A}$ is the state of the i^th^ variable's j^th^ stimulator, $x_{ik}^{I}$ is the state of the i^th^ variable's k^th^ inhibitor. The 3-state logic operators given in Equation (2) are described in further detail in Craddock et al. (Craddock et al., 2014) and provided in Tables S1–S3. The first entry in Equation (2) is used when the variable possesses stimulators and inhibitors, the middle when the variable has only stimulators, and last when the variable has only inhibitors. More directly, the target will increase when there are more stimulating signals and decrease when there are more inhibiting signals. The situation where the logic indicates that the current state prefers to evolve toward itself indicates that the current state is a stable state of the system (i.e., it does not evolve in time).

### Simulation of putative treatments

A simulation algorithm, as described previously in Craddock et al. (2015), was used to analyze the response of the neuroimmune model to external perturbations. Briefly, from any initial state, allowable state evolutions are determined based on the 3-valued logic described in Equation (2). Applying Equation (2) to each variable in the model for the m^th^ state of the system, ${\vec{x}}^{m}\left(t\right)$, defines the image vector ${\vec{x}}^{m}\left(t+1\right)$ for the m^th^ state. With ${\vec{x}}^{m}\left(t+1\right)$ defined, the system may be updated asynchronously (allowing only one variable to change at a time) following the generalized logical analysis of Thomas (1991). According to this method, the i^th^ variable of the m^th^ state vector $x_{i}^{m}\left(t\right)$ is moved one step toward its preferred image $x_{i}^{m}\left(t+1\right)$ (e.g. If $x_{i}^{m}\left(t\right)$ = −1 and $x_{i}^{m}\left(t+1\right)$ = 1, then $x_{i}^{m}\left(t+1\right)$ is set to 0). Thus, for each current state of the system there are potentially several subsequent states toward which it may asynchronously evolve. From the allowable transitions the target state is chosen at random using a uniform equal distribution and used to generate the next set of allowable target states. Stable states for which the preferred state of evolution is the same as the current state are considered stable (steady states, attractors, basins etc.), and do not evolve further in time are endpoints in the evolution of the system. This un-weighted random “Monte Carlo” procedure is performed until such a stable state is reached. Executing the simulation multiple times gives a distribution of paths that is used to determine the behavior of the system from any given initial state.

To identify a robust intervention capable of moving the neuroimmune signaling system from an alternate, potentially pathological, mode of regulation to that of typical health, putative treatment strategies were simulated. For each candidate treatment strategy, simulations were conducted to evaluate the likelihood of returning the system from an alternate stable regulatory state, to the stable state of typical health, as described previously in Craddock et al. (2015). Specifically, starting from the alternate regulatory stable state, a variable affected by the putative treatment was shifted according to the treatments mode of action: −1 (suppressing), 0 (untreated), and 1 (elevating). Following this initial perturbation, the system was allowed to evolve in time following the “Monte Carlo” procedure described above until a stable state was reached. To obtain a percentage of simulations returning to typical health, each putative treatment was run 1000 times. Due to the random nature of the “Monte Carlo” procedure, this process was performed 10 times for each treatment strategy to obtain a distribution of the likelihood for a treatment to return the system to the stable state of typical health.

### RNAseq-based gene expression analysis in stress primed mouse model of neuroinflammation

Isolated RNA collected previously from our mouse model (O'Callaghan et al., 2015) was sent to Q Squared Solutions Expression Analysis, LLC (Morrisville, NC, USA) for analysis by Illumina HiSeq/TruSeq Stranded mRNA (San Diego, CA, USA). Briefly, mice were exposed to CORT (200 mg/L in 0.6% ethanol) in the drinking water for 7 days followed by a single injection of DFP (4 mg/kg, i.p.) or saline (0.9%, vehicle) and killed by decapitation 6 h post-DFP. Brains were removed from the skull and cortex dissected free-hand for subsequent isolation of RNA, as previously described (O'Callaghan et al., 2015). RNA integrity and quantity were measured by BioAnalyzer (Santa Clara, CA, USA) and NanoDrop (ThermoFisher Scientific, Waltham, MA, USA). Normalized RNA counts were provided for saline-, CORT-, DFP-, and CORT+DFP-treated groups.

### Ethics statement

All procedures were performed under protocols approved by the Institutional Animal Care and Use Committee of the Centers for Disease Control and Prevention, National Institute for Occupational Safety and Health, and the animal facility was certified by the American Association for Accreditation of Laboratory Animal Care (CDC NIOSH HELD ACUC (CDC) cdcnioshheldacuc@cdc.gov, Protocol # 17-004 v3).

### Comparison of gene expression data to model predicted states

In an effort to check the validity of our model, we compared stable states predicted by the latter to gene expression profiles measured experimentally in a mouse model of Gulf War Illness (O'Callaghan et al., 2015). Brown's theoretical approximation (Brown, 1975) of Fisher's statistics was used as a measure of similarity between a given model predicted state and the expression profile measured in a subset of genes corresponding to the cellular and molecular entities used in our model, as done in our previous work (Craddock et al., 2014, 2015; Fritsch et al., 2014; Rice et al., 2014). Fisher's statistics provide a meta-analysis technique to combine probabilities and obtain the overall significance of a set of *p*-values corresponding to independent tests of the same null hypothesis. The combined χ^2^ statistic,

(3) $$\begin{array}{r} T_{0}=-2\overset{N}{\underset{i=1}{\sum }}\ln\left(p_{i}\right) \end{array}$$

where N is the number of measureable variables and p_i_ are the corresponding *p*-values under the null hypothesis, has a χ^2^ distribution with 2N degrees of freedom assuming the hypothesis tests are independent. As evidenced by the connectivity of the system studied here, these model entities do not express independently. As a result, direct application of this test statistic is not valid since the assumption of independence is violated. To accommodate this Brown (1975) suggested a method for combining non-independent tests. If the tests are not independent, then the statistic T_0_ has mean m = 2N and variance (σ^2^) given as,

(4) $$\begin{array}{r} \sigma^{2}=4N+2\overset{N-1}{\underset{i=1}{\sum }}\overset{N}{\underset{j=i+1}{\sum }}cov\left(-2lnp_{i},-2lnp_{j}\right) \end{array}$$

where p_i_ and p_j_ are the *p*-values for each test and the covariance (cov) is calculated as,

(5) $$\begin{array}{l} cov\left(-2lnp_{i},-2lnp_{j}\right)\\ =\{\begin{array}{c} \rho_{ij}\left(3.25+0.75\rho_{ij}\right),0\leq \rho_{ij}\leq 1\\ \rho_{ij}\left(3.27+0.71\rho_{ij}\right),-0.5\leq \rho_{ij}<0 \end{array} \end{array}$$

with ρ_ij_ being the unadulterated correlation between variable i and variable j. Finally, the overall significance P of a set of non-independent tests is calculated using the statistic T which under the null hypothesis follows the central χ^2^ distribution, where T = T_0_/c with 2N/c degrees of freedom and c = σ^2^/4N.

Here, we test if the expression levels of the subset of genes corresponding to the cellular and molecular entities used in our model align with a given model predicted discrete state profile. Our null hypothesis is that the experimental measures do not align with model predictions of greater than control (normal), lower than control or in alignment with control levels. First, *p*-values for individual variables, p_i_, are calculated using two-sample *t*-tests comparing expression levels in ill subjects with those measured in healthy controls. Where the model predicts marker expression to be high (+1), a ‘right-handed’ one-tailed test is used to confirm that measured expression levels are significantly greater than the reference control (0). Conversely a ‘left-handed’ test is used when the model predicts a low (−1) to confirm that the measured expression levels are on average significantly lower than the reference control (0). For the case where the model predicts normal expression levels for a variable (0), a two-tailed *t*-test is used. However, the *p*-value from the two-tailed test, p_two−tail_, gives the probability that there is an observable difference between illness and control, which is the null hypothesis. To rectify this, when comparing to a model predicted variable of 0 we take the *p*-value to be p_i_ = 1 - p_two−tail_, giving the probability of obtaining the predicted value when the null hypothesis is true. The unadulterated correlation values ρ_ij_ between two variables i and j were calculated in saline treated animals as the pairwise Pearson's linear correlation coefficient between variables.

The above-mentioned experimental data were compared against model predictions based on 12 measured variables (Table S4), including 7 signaling molecules, namely brain derived neurotrophic factor (BDNF), insulin like growth factor 1 (IGF-1), interleukin (IL)-1β, IL-6, IL-4 tumor necrosis factor (TNF) α, CD200, and vascular endothelial growth factor A (VEGFA). Markers for 2 cell types were also included in the comparison with astrocyte status measured by glial fibrillary acidic protein (GFAP), and microglia activation measured by leukemia inhibitory factor (LIF), oncostatin M (OSM), and chemokine (C-C motif) ligand 2 (CCL2), which positively correlate with cellular activity. Where model variables represent an aggregate set of markers, each experimentally measured constituent marker was compared individually to the model predicted value. For example, microglia activation was determined by LIF, OSM, and CCL2; therefore, 3 individual *p*-values were calculated based on the predicted value of microglia.

A comparison of the measured exposure response states with the model predicted stable states is best visualized by projecting these 12-dimensional co-expression profiles into a two-dimensional space using a non-parametric multidimensional scaling. Specifically, Sammon's nonlinear mapping criterion (Sammon, 1969) was used to project the *p*-value distances onto a 2-dimensional plot and describe the statistical significance of separation between measured and predicted co-expression patterns. Aggregate *p*-values between predicted model states were determined via Brown's method above, where values between predicted states that were found to disagree with *P*-values for individual variables, p_i_, were taken as 1. Conversely, when values between predicted states were found to agree, p_i_ was assigned a standard minimum value for significance of 0.05 to avoid numerical instability in the calculation of the combined χ^2^ statistic T_0_.

While the aggregate *p*-value provides a measure of significance to the alignment of experimental data with multiple model predicted states, it does not quantify the nature or extent of this alignment. To obtain a quantitative measure of the degree of the agreement between the measured response and the predicted stable state, the fold change with respect to resting reference state levels was calculated across all measures. As the fold change value for an individual measure can be either positive or negative depending on whether the effect causes an increase or decrease in expression compared to saline control, it can be compared directly to the model-predicted directions of change.

## Results

### Literature based discrete neuro-immune signaling network

**Figure 1:**
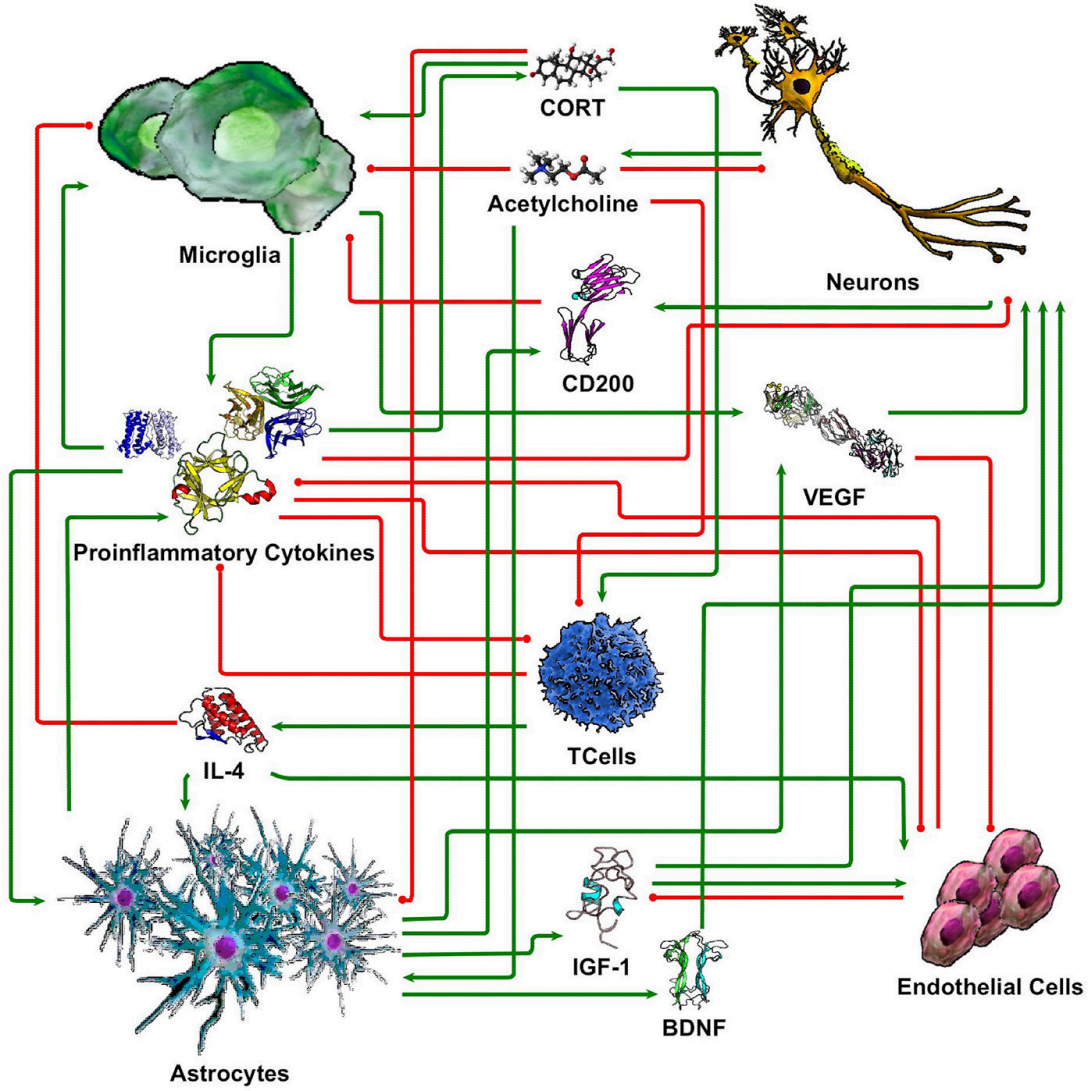
Simple neuroimmune signaling network. Connections with green arrow terminators represent stimulatory effects while connections with red circle terminators represent inhibitory effects.

While our understanding of neuronal-glial interactions continues to grow, we present here a minimal model of the principal regulatory mechanisms linking 5 cell types (neurons, microglia, astrocytes, endothelial cells, and T cells), through 10 signaling molecules (cortisol/corticosterone, acetylcholine, CD200, IL-1β, IL-4, IL-6, TNF-α, IGF-1, VEGF, and BDNF). These were chosen based on their prevalence in the literature and their perceived importance in neuronal-glial interaction. While this limited set by no means fully accommodates the vast complexity of the neuroimmune system, the corresponding coarse-grained circuit model supports a first approximation of physiologically relevant and experimentally verifiable response dynamics. The basic literature informed structure of this this signaling network is illustrated in Figure 1. In the following section, we describe each of these signaling connections with references to the supporting literature.

### Microglia

Microglia are immune cells residing within the CNS that are actively involved in monitoring activity in the CNS and even minor changes or dysregulation of microglia can lead to neuropathological effects (Kierdorf and Prinz, 2013). Upon activation, microglia have a heterogenic multistage activation process that allows them to secrete numerous cytokines and growth factors and influence various cell types within the CNS (Kettenmann et al., 2011; Jurgens and Johnson, 2012; Jha et al., 2013). In the absence of disease or external triggering factors, microglia exist in a quiescent “resting” state with cellular extensions or ramified processes continuously extending to and retracting from surrounding neural tissues, constantly surveying their surroundings for pathogens (Davalos et al., 2005; Nimmerjahn et al., 2005). Activating signals from infection, trauma, or cell impairment trigger a transition of microglia to distinct reactive phenotypes accompanied by an increase in size, the release of cytokines and chemokines, and phagocytic activity (Hanisch, 2002). Microglial dysfunction, on the other hand, is characterized by dystrophy and degeneration of microglial cells as evidenced by fragmentation, loss of fine processes, and swellings at the terminals of cytoplasmic processes often observed in aging (Damani et al., 2011; Streit et al., 2014). Therefore, we model microglia as typically resting, with elevation representing an increased number of activated microglia, and depression as representing an increased number of dysfunctional microglia.

Factors that are known to stimulate microglia include increased glucocorticoid levels accompanying stress, as well as proinflammatory cytokines IL-1β, IL-6, and TNFα. Exogenous glucocorticoids have been shown to “prime” microglia to have a potentiated response after repeated inescapable shocks in rat models (Frank et al., 2007, 2012) and have been shown to increase the inflammatory response to organophosphate exposure in mice (O'Callaghan et al., 2015; Locker et al., 2017). The proinflammatory cytokines TNFα, classical IL-1 family members, and IL-6 have all been shown to both activate microglia and be produced by activated microglia (Hanisch, 2002). Since microglia are activated by, and have the ability to secrete proinflammatory cytokines (IL-1, IL-6, TNF-α) once activated, this creates a positive feedback mechanism (Jurgens and Johnson, 2012; Burton et al., 2013). Thus, these signals have the potential to produce continual activation of microglia and are, therefore, critical for both the protective actions as well as harmful outcomes of microglial engagement.

Microglia activity is reported to decrease through the actions of IL-4, CD200, and acetylcholine. It has been shown that IL-4 reverses the inflammatory toxicity caused by activated microglia (Butovsky et al., 2006), and the absence of IL-4 heightens vulnerability to neuroinflammation (Gadani et al., 2012). CD200 is expressed in several cell types including neurons (Webb and Barclay, 1984; Lyons et al., 2007) and astrocytes (Costello et al., 2011) and CD200-deficiency has been associated with increased microglial activation accompanied by increased production of inflammatory cytokines (Denieffe et al., 2013). In the absence of the regulatory action of CD200, microglia and infiltrating immune cells can cause injury as well as adverse inflammatory responses in either acute or chronic conditions (Hernangómez et al., 2014). As such, CD200 plays a key role in immune responses in health and disease. Microglial secretion of pro-inflammatory cytokines is considered inhibited by cholinergic neurotransmission. For example, increasing cholinergic activity with cholinesterase inhibitors suppresses microglial release of TNFα and IL1β following LPS exposure (Tyagi et al., 2010). Cholinergic input to microglia cells, particular via nicotinic acetylcholine receptors, has been shown to limit microglia-mediated neurotoxicity *in-vivo, in-vitro*, and in ex-plant cultures (Wang et al., 2003; Shytle et al., 2004; Carnevale et al., 2007; Kawamata and Shimohama, 2011; Kalb et al., 2013). Peripherally administrated acetylcholinesterase inhibitors reduce brain and blood IL-1β production (Pollak et al., 2005).

### Astrocytes

Astrocytes, the most numerous cell-type in the vertebrate CNS, are a sub-type of glial cell that perform various functions, some of which include providing structural support for neuronal elements, regulating extracellular glutamate, the secretion of growth factors and cytokines, and interacting with endothelial cells to reinforce the blood-brain barrier (BBB)(Sofroniew, 2000). Astrocytes persist in an inactive/quiescent state performing the majority of the functions listed above. In response to CNS trauma, via physical (traumatic brain injury), chemical, or biological (infection or disease) means, astrocytes can become activated and reactive (Pekny and Nilsson, 2005; Sriram and O'Callaghan, 2005; Jha et al., 2013), changing their function and morphology in a heterogenic manner (Zamanian et al., 2012). Known as gliosis, astrogliosis, or reactive gliosis, such hypertrophic changes have been demonstrated in hormonal, neuropathological, and environmental conditions (Safavi-Abbasi et al., 2001; Faulkner et al., 2004; Pekny and Nilsson, 2005; Torres-Platas et al., 2011). There is also increasing evidence of astrocytic atrophy in chronic disease (Lechuga-Sancho et al., 2006; Tynan et al., 2010). As such, we model astrocytes as typically resting, with an elevated expression state representing an increased number of reactive astrocytes, and depressed expression state representing astrocyte atrophy or an inhibition of astrocyte proliferation.

During inflammation, astrocytes are producers of a variety of cytokines including IL-1, IL-6, and TNF-α (Dong and Benveniste, 2001). Active astrocytes provide a neuroprotective role through the production of IGF-1 and BDNF (Fulmer et al., 2014; Sofroniew, 2014). In addition, astrocytes stimulated by IL-1 signaling will secrete VEGF (John et al., 2005). Furthermore, the proinflammatory cytokines TNF-α and IL-6 induce astrocyte proliferation (Selmaj et al., 1990). IL-4 enhances astrocytic secretion of BDNF *in-vitro*, as demonstrated in animal models (Derecki et al., 2010). Moreover, BDNF expression is increased in mice undergoing spatial memory training, an effect not seen in IL-4 deficient mice (Derecki et al., 2010).

### Neurons

Neurons, the electrically excitable cells that process and transmit electrical and chemical signals, are the core components of the CNS. Neuroscience views complex cognitive functions, for example learning, memory, decision-making, as well as the influence of emotion and motivation upon cognition, as emerging naturally from the complex electrical (Nichols and Newsome, 1999), and chemical activity of large systems of neurons within the brain. Thus, in our model, neurons are representative of both neuronal health and cognitive function with an elevated state representing an increased number of active neurons involved in cognitive activity (learning, memory formation or recall, active attention etc.) and decreased functional state indicating an increased number of inactive neurons and cognitive deficit. While this is a very coarse representation of the complexity of neuronal function within the brain, it nonetheless offers a useful abstract representation of broad response behavior.

Healthy neurons maintain microglia in their resting state via secreted and membrane bound signals including CD200, neurotransmitters and neurotrophins such as BDNF (Pocock and Kettenmann, 2007). A reduction in these regulatory factors can lead to a reactive microglia phenotype, which chronically could lead to neuronal death, suggesting an important role for communication between neurons and microglia in regulating neuroinflammation (Jurgens and Johnson, 2012). Upregulation of IL-1β and TNF-α is a common feature of glial cell activation in neuroinflammatory conditions (Hu et al., 1995; Ye et al., 2013), and IL-1β and TNF-α alone or in combination with other cytokines can promote neuronal death and apoptosis (Chao et al., 1995; Bender et al., 2005; Ye et al., 2013).

It has been well established that aging is associated with elevated serum IL-6 levels, an observation that correlates with age-related cognitive decline. Genetic ablation of IL-6 in mice yields improved memory formation as IL-6 knockout mice were less sensitive to scopolamine-induced amnesia (Braida et al., 2004). Both chronic and acute blockade of IL-6 signaling are associated with improved memory (Balschun et al., 2004). Neurons are also influenced by proinflammatory cytokine IL-1β, which directly interferes with both BDNF and NT-3 signaling pathways, suggesting the immune neurochemical pathways (Soiampornkul et al., 2008; Tong et al., 2008; Jurgens and Johnson, 2012). Furthermore, *in vivo*, neuronal stimulating and growth factors such as BDNF, NGF, and NT-3 protein levels were significantly reduced (20–40%) in the hippocampus and cortex for 7 days following peripheral LPS injection in rats, suggesting the role of immune challenge in neuronal health (Guan and Fang, 2006). Overall, neuronal dysfunction and subsequently cognitive decline is perpetuated by inflammatory cytokines, such as IL-1β, IL-6 and TNF-α. Thus, here we model the pro-inflammatory cytokines IL-1β, IL-6, and TNF-α as inhibiting normal neuron function (Figure 1).

Factors that have been shown to increase neuron activity include acetylcholine and growth factors including BDNF, VEGF, and IGF-1. Acetylcholine is a key neurotransmitter that is known to facilitate long-term potentiation (LTP), thereby promoting learning and memory (Li et al., 2000). The growth factor, VEGF has been shown to promote proliferation of cortical neuron precursors (Zhu et al., 2003). BDNF has been shown to promote neuronal health by both synaptic and dendrite spine formation, and inhibition of BDNF conversely leads to neuronal dysfunction (Lin and Koleske, 2010). IGF-1 has been shown to be involved in memory, plasticity, neurogenic processes, and in neuronal rescue post-insult, while deficiency in IGF-1 results in decreased memory and processing speeds (Sonntag et al., 2005).

### Endothelial cells

Endothelial cells form a thin layer that lines the interior surface of blood vessels creating an interface, known as the BBB, between circulating blood from the periphery and the extracellular fluid of the brain. The integrity of the BBB relies on the tight junctions between endothelial cells separating the CNS from the other systems. In this model, endothelial cells represent the integrity of the BBB. Inhibition of endothelial cells leads to leaky membranes, while promotion suggests strengthening of the BBB and decreased permeability.

Various stimuli, such as hypoxia or inflammation, have the ability to weaken the integrity of the tight junctions (TJs) on endothelial cells (Abbott et al., 2006; Pan et al., 2011), while others, like IGF-1, can significantly reduce BBB permeability (Bake et al., 2014). Changes to the integrity of the BBB can also reciprocally cause increases in these same soluble messengers. Endothelial cells under oxygen-glucose deprivation, in a cell culture model of ischemia, secrete IGF-1 (Wang et al., 2013). Likewise, proinflammatory cytokines, such as IL-1, IL-6, and TNF-α, which are transported across the BBB by distinctive unidirectional and saturable transport systems, increase with BBB disruption; however, the exact mechanisms are not fully understood (Erickson et al., 2012).

### T cells

Historically, the brain has been considered an immunologically privileged site to which access of circulating immune cells is tightly controlled by the endothelial BBB. However, it now appears that T Cells can enter the brain in small numbers and communicate with resident cells of the CNS under certain conditions (Larochelle et al., 2011; Yirmiya and Goshen, 2011; Engelhardt and Ransohoff, 2012). More specifically, T cell play a role within the brain via involvement in continual surveillance and neurogenesis which is very important in learning and memory and as demonstrated by experiments by Kipnis et al. (Kipnis et al., 2004) and Ellwardt et al. (Ellwardt et al., 2016). Proinflammatory cytokine signals (IL-1B, IL-6, and TNF-α) can serve to prime T cells toward a Th1 type immune response, and away from a Th2 type response. The Th2 type response is responsible for the production of anti-inflammatory cytokines, including IL-4, which has important anti-inflammatory and immunosuppressive activities, which serves to inhibit inflammation. While it is known that T Cells produce and respond to both cytokine and neurotransmitter signals, the details of these signaling processes are not completely understood at this time. Here, we include basic T Cells in our model as they have been shown to be key to both learning and immunity. The T Cell state in the model broadly represents the Th2/Th1 profile ratio with elevation corresponding to Th2 anti-inflammatory activity, depression to Th1 inflammatory activity, and nominal referring to an undifferentiated T Cell.

### Hypothalamic-pituitary-adrenal axis and glucocorticoids

The hypothalamic–pituitary–adrenal (HPA) axis orchestrates the systemic release of glucocorticoids in response to physiological and psychogenic stressors. Elevated brain cytokines are thought to produce activation of stress response systems such as the hypothalamic-pituitary-adrenal (HPA) axis with the subsequent secretion of the species-specific glucocorticoid from the adrenal glands (Dunn, 2000; Mizoguchi et al., 2000; Yirmiya and Goshen, 2011; Hinwood et al., 2012; Shansky and Lipps, 2013). Pro-inflammatory cytokines in general, and IL-1β in specific, are essential mediator of the stress response (Goshen and Yirmiya, 2009). IL-6 has been found to induce a stress response in the HPA axis in the absence of CRH (Bethin et al., 2000) and this positive feed-forward loop may function to perpetuate an inflammatory state within the CNS resulting in excitotoxicity (Tikka et al., 2001). Furthermore, under chronic, severe stress, sustained activation of the HPA axis results in learning and memory impairments (McEwen and Sapolsky, 1995; De Kloet, 2004; De Kloet et al., 2005).

Glucocorticoids appear to have a biphasic effect on neurons and glial cells (Miller and O'Callaghan, 2003, 2005; Jauregui-Huerta et al., 2010), with high stress levels being pro-inflammatory, while basal or low stress levels have traditional anti-inflammatory effects. However, exogenous corticosteroids have been shown to induce activation of microglia, the major immune cell in the CNS (Frank et al., 2007, 2012; Jurgens and Johnson, 2012), as well as proliferation (Nair and Bonneau, 2006) and morphological transformation of microglia from a resting to an activated state (Jauregui-Huerta et al., 2010; Tynan et al., 2010).

Experiments in rat models have shown that chronic stress results in depleted astrogliogenesis (Jauregui-Huerta et al., 2010), and *in vitro* studies found that glucocorticoid selectively blocks spontaneous astrogliogenesis from neural precursor cells (Sabolek et al., 2006). Based on these findings, it can be proposed that stressors induce a reduction of astroglia (Miller and O'Callaghan, 2003, 2005; Jauregui-Huerta et al., 2010). Additionally, the stress response itself causes reversible structural remodeling of medial prefrontal cortical neurons (McEwen et al., 2016).

Overall, data suggests that stressors can shift the neuroimmune microenvironment toward a pro-inflammatory state, resulting in an exaggerated response upon additional immune stimulation as well as detrimental effects on regulatory molecular pathways and neural circuits (Jurgens and Johnson, 2012).

### Persistent states of the neuro-immune model

**Figure 2:**
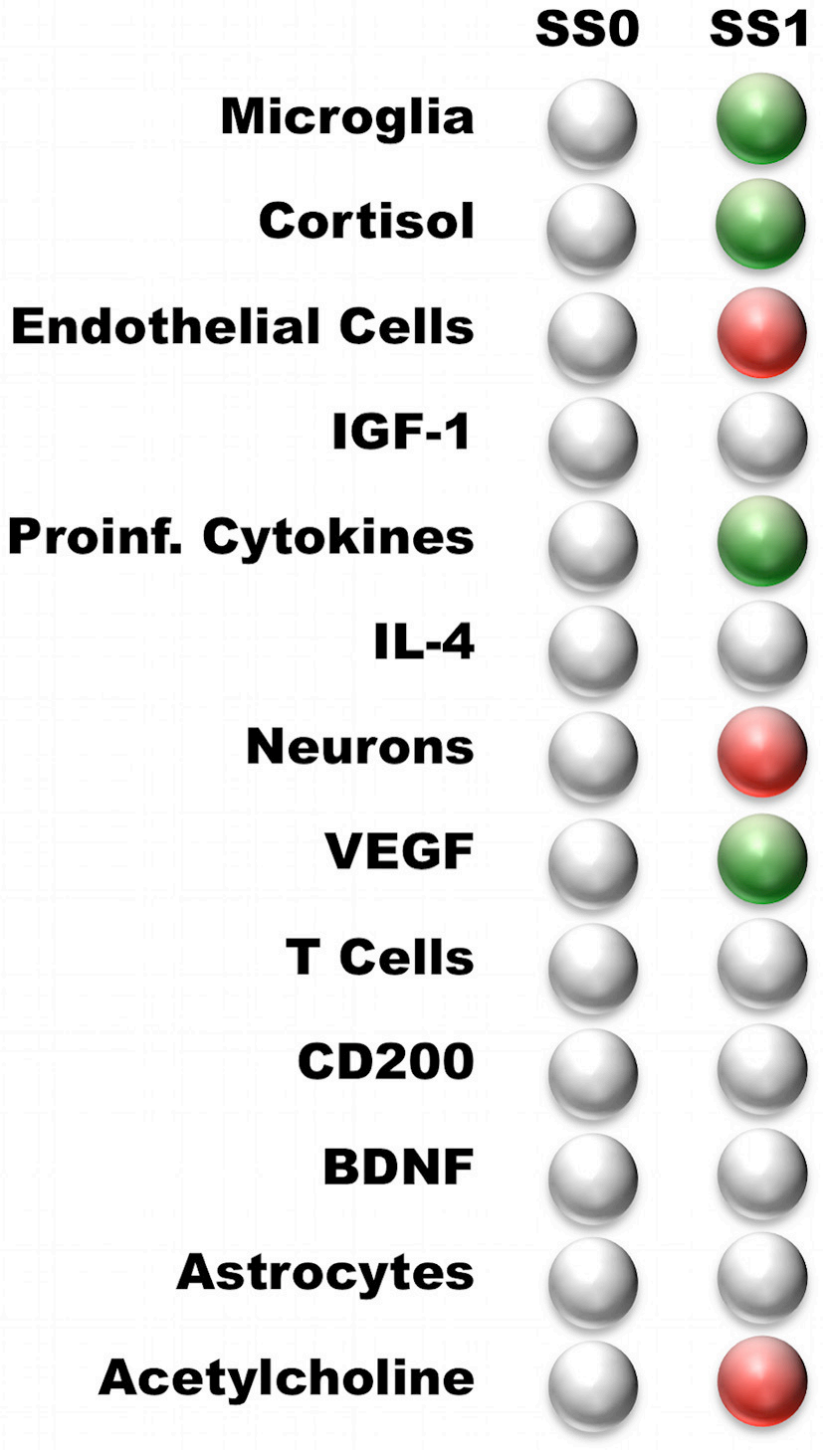
Steady states predicted by the neuro-immune model. White—nominal state (0); Green—high state (1); Red—low state (−1). The steady states are named according to their profile: Typical Health, and Neuroinflammation. Typical Health state is shown for reference.

The neuroimmune signaling network portrayed in Figure 1 has 13 molecular and cellular components that may each take 1 of 3 potential values (low, typical, or high). Since the number of states in the system scales exponentially with the number of components, there are approximately 1.6 million potential states available to the system (3^13^ = 1,594,323). Analysis of the neuroimmune signaling network using the discrete 3-valued logic described in the Methods revealed two persistent homeostatic behaviors (Figure 2). In the first persistent behavior (SS0), all cellular and molecular variables were found to express at baseline values, which is representative of typical healthy neuroimmune regulation. The alternate steady state (SS1) is characterized by high levels of the stress hormone CORT, the pro-inflammatory cytokines IL-1β, IL-6, and TNFα, the growth factor VEGF and microglia activation, with depressed levels of acetylcholine, reduced neuron function, and an increased BBB permeability. This alternate state represents a stable self-sustaining pathological neuroinflammatory regime that persists even in the absence of an external aggravating factor and is different from typical physiological neuroinflammation (i.e., due to minor infection), which resolves over time, and would be represented as a transient condition in this model.

### Arrangement of neuro-inflammatory conditions in the neuro-immune state space

**Figure 3:**
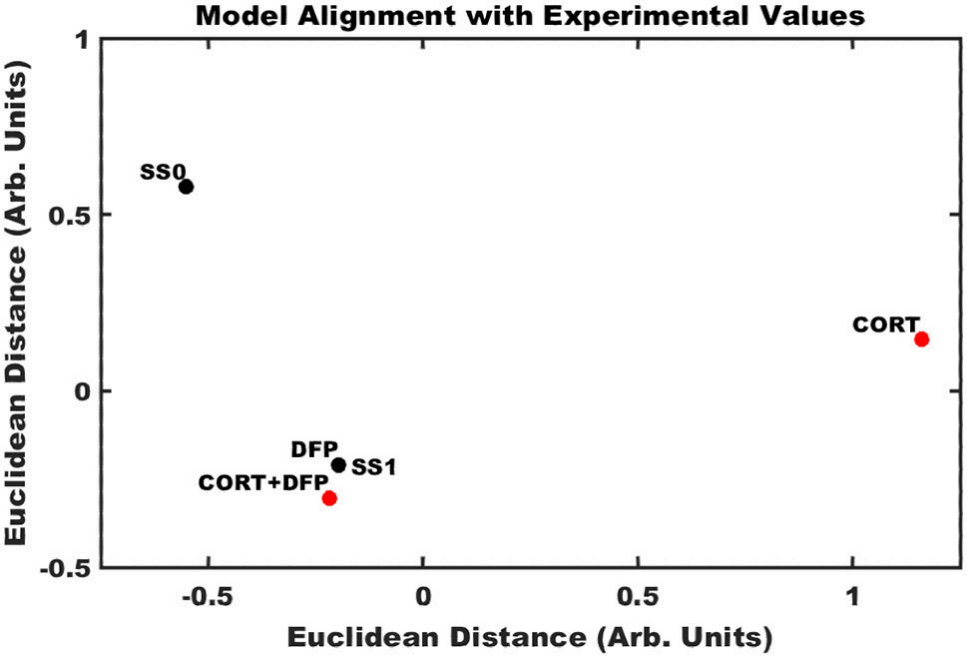
Alignment of exposure conditions compared to saline controls with model predicted stable states. Sammon projection in two dimensions for gene expression obtained in cortex. Black dots represent the model predicted stable states (SS0, and SS1). Red dots represent the aggregated gene expression data for CORT, DFP, and CORT + DFP exposure conditions vs. saline controls. Axes represent arbitrary units such that the relative distance between points approximates the aggregated *P*-values between all points.

Application of the Brown's meta-analysis method described above allowed for the calculation of an aggregate *p*-value simultaneously comparing the ensemble of experimentally measured expression profiles (Table S4) with the model-predicted stable states. Projecting these 12-dimensioanl profiles into a two-dimensional space allowed for the comparison of multiple illness conditions in the landscape surrounding the two predicted stable states (Figure 3). In the cortex, it was found that DFP and CORT+DFP exposure models more closely resembled the alternate SS1 state of persistent neuroinflammation (*p* = 0.0002 and 0.02, respectively) than the typical state SS0 (*p* = 0.99 and 0.94, respectively). Exposure to CORT alone did not align well with the neuroinflammatory state SS1 (*p* = 0.43), but similarly did not align with the normal typical neuroimmune regulatory state SS0 (*p* = 0.94). This suggests that CORT exposure perturbs the neuroimmune system and places it in a transient state removed from both of the persistent regulatory modes available, but which is also capable of moving toward either of these modes depending on available conditions or subsequent exposures.

**Figure 4:**
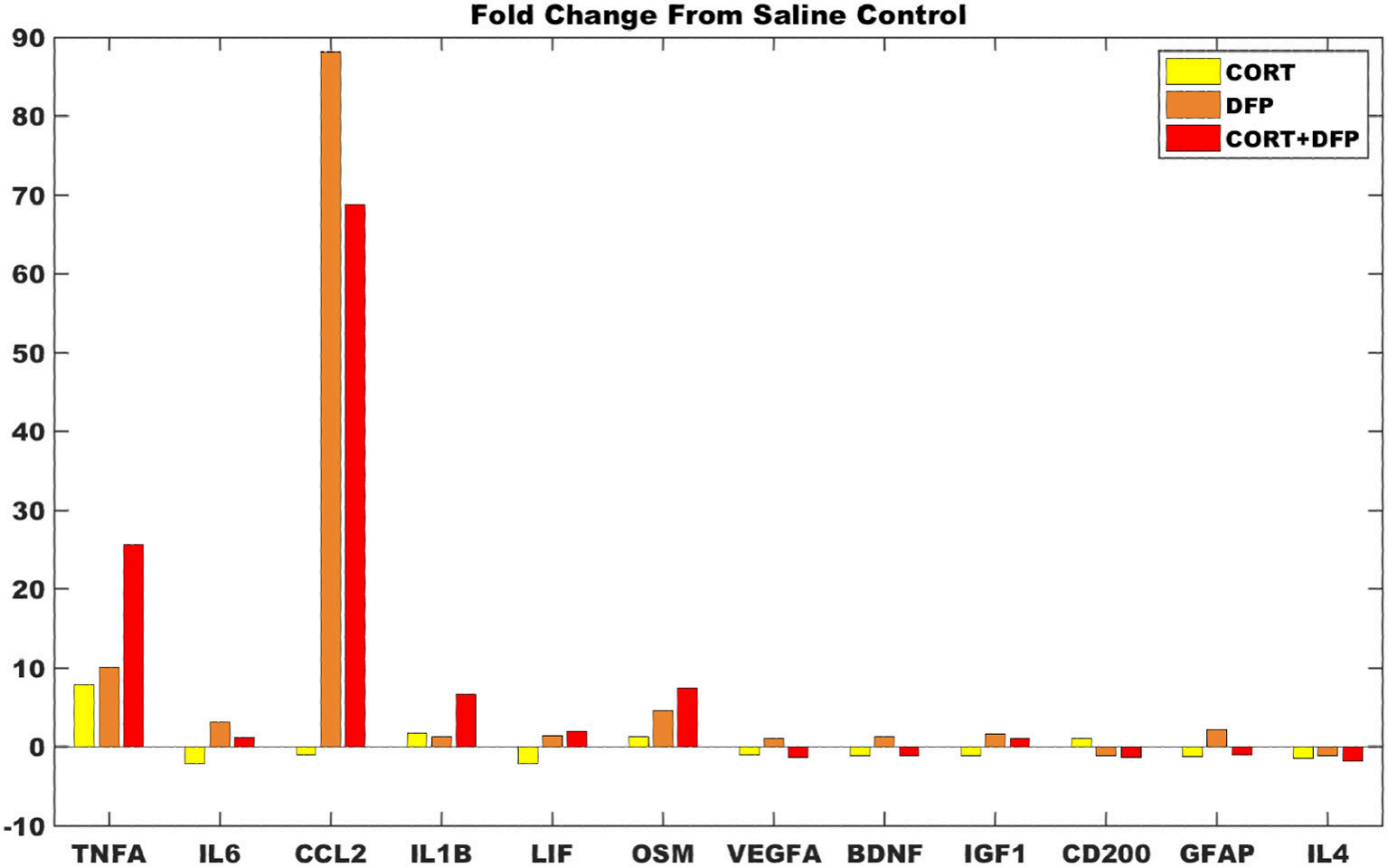
Gene expression changes in mouse cortex for exposure conditions compared to control. Fold change in marker gene expression levels from RNAseq of samples collected at 6 h relative to resting saline control for data obtained in cortex for the subset of 12 markers represented in the logic circuit model.

For the majority of measures from the DFP and CORT+DFP fold changes are consistent with the model predictions (Figure 4), save VEGF which was predicted to have be overexpressed but presents with only a small fold change. In the cortex, CORT exposure alone was found to produce a negligible neuroinflammatory effect. However, for both the DFP and CORT+DFP exposures, the large fold changes across measures of proinflammatory cytokines and microglia activations was found indicative of a neuroinflammatory effect. In the cortex, the CORT+DFP condition resulted in slightly larger effects than DFP alone except in the measure of microglia activity by CCL2. The most prominent neuroinflammatory effect for both DFP and CORT+DFP is seen in CCL2 (microglia) and TNFα.

### Simulated treatment strategies

**Figure 5:**
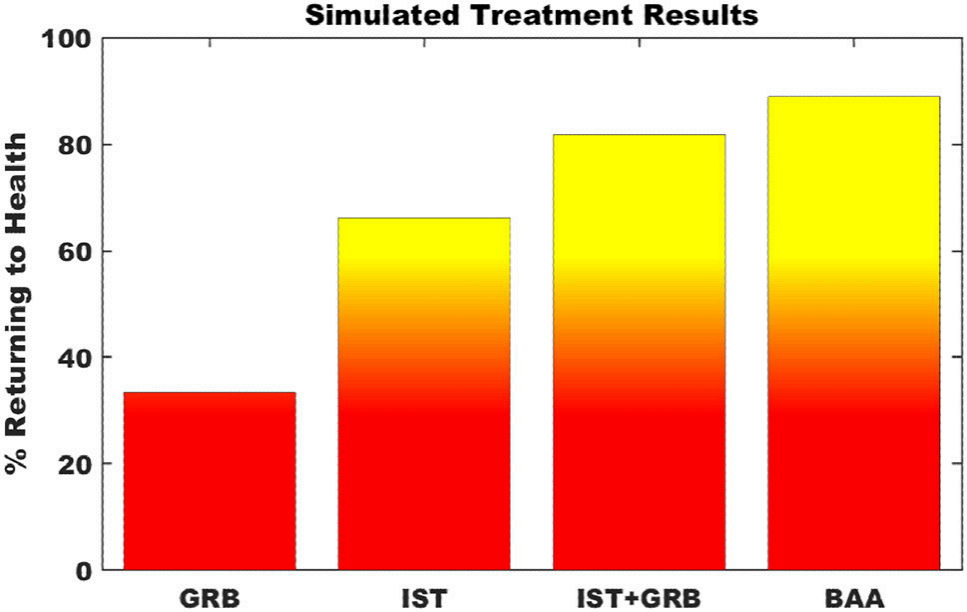
Simulation of putative treatment strategies. Percent of Monte Carlo simulations of putative treatment strategies that return the neuroimmune system to typically healthy regulation. GRB—glucocorticoid receptor blockade; IST—immunosuppressive therapy; BAA—broad acting anti-inflammatory. Transition to yellow indicates point of greater than 50% of trials returning to health.

Simulating single point interventions that perturb the persistent neuroinflammtory states of the neuroimmune model allowed for the testing of putative treatment strategies supporting a return of the system to typical healthy regulation. A focused subset of candidate strategies was chosen that specifically addressed or were relevant to the markers predicted to be dysregulated at the neuroinflammatory state, namely elevated levels of CORT, proinflammatory cytokines, and microglial activity. Specifically, these candidate strategies involved the minimization of downstream effects of elevated CORT through blockade of the glucocorticoid receptor (GRB) by antagonists such as mifepristone, immunosuppresive therapy (IST) aimed to specifically inhibit proinflammatory cytokines IL-1β, IL-6, and TNFα via biological response modifiers such as anakinra, canakinumab, sirukumab, etanercept, or infliximab, and inhibition of microglial activation and simultaneous down regulation of pro-inflammatory cytokine output via a broad acting anti-inflammatory agent (BAA), (e.g., propranolol or tetracyclines such as minocycline). Additionally, a combination of GRB and IST for simultaneously decreasing both elevated CORT and the proinflammatory cytokines was also simulated. Figure 5 presents the results of these simulated interventions. Success of the treatments, measured as the frequency with which the model neuro-immune system was rescued from its stable inflammatory cascade, suggest that BAA is the single most effective agent, returning 88.9% of simulations to health. This is followed by combination therapy IST+GRB (81.8%), IST (66.3%), and GRB (33.4%).

## Discussion

Neuroimmune signaling is highly complex. Communication between neuronal and glial cells is mediated by neurotransmitter, hormone and cytokines levels in the brain, which are further regulated by the BBB and T Cells within the CNS (Tian et al., 2012). Alterations in this web of communication are essential to the neuroimmune response to physical, chemical, or biological insult. However, significant irregularities in these signaling patterns can lead to negative impacts on brain function including learning and memory. We hypothesized that the complexity of the neuroimmune signaling network can allow for multiple regulatory modes beyond what is typically considered normal health and that the chronic neuroinflammation seen in disorders of the brain are perpetuated, at least in part, by such naturally occurring homeostatic regulatory programming.

Using a discrete logic methodology proposed by Thomas (1991) and further developed by Mendoza and Xenarios (2006) as well as our group (Craddock et al., 2014), we demonstrated that the naturally occurring feed-forward and feedback signals of the neuroimmune system allow for alternate regulatory behaviors. The simple model proposed in this paper captures the basic signaling between different type of cells in the neuroimmune response via various cytokines, signaling molecules, hormones, and growth factors in response to psychological stressors activating the HPA axis. Furthermore, this model may be used to simulate the effect of pharmaceuticals or toxins on neuro-immune signaling via modulation of molecular markers affected by the chemicals in question. Throughout the model, feedback loops, both positive and negative, have been implemented and appear prominently in microglial signaling, an important contributor to the perpetuation of neuroinflammation. The model demonstrates that these positive feedback loops are central to the appearance of an alternate regulatory mode. Importantly, the alternate homeostatic mode predicted by the model is characterized as having one of chronic neuroinflammation (high levels of cortisol/corticosterone, and pro-inflammatory cytokines, microglia activation, depressed levels of acetylcholine, IL-4, VEGFA, reduced neuron function, T Cells in a Th1 profile, and increased BBB permeability). It must be noted that this alternate mode emerges as a direct result of the signaling connectivity documented in the literature, in particular, the presence of positive feedback loops.

Moreover, we emphasize that the alternate regulatory mode predicted here is naturally supported by a normal and intact immune signaling network and may exist to provide medium or long-term survival through what becomes, essentially, an adaptive compromise in the wake of an insult. As such, it supports the premise that neuroinflammation can occur without neuronal degeneration or other damage to the circuit (O'Callaghan et al., 2015). While prolonged neuroinflammation can involve a degenerative component or functional compromise of specific signaling components, such long-term damage was not modeled here. As we expect that the continued presence of an aggravating factor (biological, chemical or physical), would result in new patterns of regulation, capturing such changes would also involve structural modifications to the computational model. While we have studied such effects in a broader model of sex hormone, stress hormone, and immune regulatory physiology (Rice et al., 2014) in Gulf War Illness one of the objectives here was to demonstrate that indeed no such changes to the signaling circuitry are required to support a persistent departure from normal neuro-immune regulation.

Comparison of the model predicted states to RNAseq gene expression profiles in a stress primed organophosphate-induced mouse model of neuroinflammation show consistency between the computational model and *in-vivo* experiment. Specifically, RNAseq gene expression profiles in cortex from mice exposed to DFP in both the absence and presence of priming by corticosterone show a greater agreement with the model predicted neuroinflammatory state as measured by Brown's meta-analysis aggregate *p*-value (*p* = 0.0002 and 0.02, respectively for the SS1 neuroinflammatory state, and *p* = 0.99 and 0.94, respectively, for the SS0 typical state). Exposure to corticosterone alone in the absence of exposure to DFP did not align well with either the model predicted neuroinflammatory state or the regulatory state of typical health suggesting that CORT priming may perturb the neuroimmune system and place it in a transient state capable of moving toward either of these modes. Estimates of the degree of neuroinflammation, as determined by the average fold change in comparison to the model predicted neuroinflammatory state, indicate that in the cortex CORT priming alone was found to produce a negligible neuroinflammatory effect, while exposure to DFP in both the absence and presence of CORT priming resulted in significant neuroinflammatory responses.

Simulation of putative treatment strategies aimed at reducing elevated levels of microglial activity, proinflammatory cytokines, and glucocorticoid levels suggested that all methods showed some degree of success. Nonetheless, the simultaneous reduction of microglial activity and proinflammatory cytokines by BAA agents was the most successful, delivering lasting stable remission in approximately 90% of computer simulated trials. This was followed closely by an 80% success rate predicted for the simultaneous reduction of proinflammatory cytokines and glucocorticoid levels. The results of these treatments are encouraging and supported by experimental work showing that an example BAA repressing microglia activation and TNFα inhibitors can reduce neuroinflammation (Sriram et al., 2006; Henry et al., 2008; Chio et al., 2013; Ye et al., 2014; Cheng et al., 2015; Dadsetan et al., 2016). Nevertheless, this model currently does not account for detailed kinetics, as data describing the magnitude and transition time of interactions between elements of the extended neuroendocrine-immune system are not available. Refinement of this model by parameters obtained from data-driven analysis will serve to improve simulations and reliability of results. Ultimately, even with these refinements, safety and efficacy of these predicted strategies must be determined experimentally.

As this model is theoretical in nature, and based on currently documented knowledge of physiology and regulatory biochemistry of a select few signaling molecules and cell types it is necessarily incomplete. Yet, while this remains a coarse grained neuroimmune model and by no means constitutes an exhaustive description, we have made every attempt to capture the primary mode of signaling and core cell behaviors. The signaling network presented here represents a base universal neuronal-glial signaling system, and is therefore disease in-specific. More specific disease context may be added in future as additional layers on top of this base disease in-specific network to provide more nuance, such as to capture the chronic inflammation and neurodegeneration seen in Alzheimer's or Parkinson's disease, or the acute inflammation seen during stroke.

Ultimately, the validity of such a model depends on its ability to replicate *in vivo* results. As this model was constructed from literature, the post-construction comparison of model predictions to data is required. While there is publically available data for studies regarding changes in gene expression and cytokine profile from human patients suffering stroke, Alzheimer's Disease, Parkinson's Disease and others, this data is generally drawn from blood, or post-mortem brain, and not directly from fresh brain tissue, questioning its ability to represent the actual neuroinflammatory brain state. As such, the mouse model of neuro-inflammation used here provides a test and validation of the model's ability to capture the neuroinflammatory response, at least in transience, for an exposure to an organophosphate in the presence and absence of stress. While, admittedly there are gross differences in brain anatomy, immune system and physiology between rodents and humans on a whole, at the level of resolution used for our coarse grained model these differences are negligible. Expanded studies examining the long-term chronicity of the neuroinflammatory state produced by such exposures in mouse models may be used to further test the model. Additionally, the predictions of several methods of alleviating this neuroinflammatory response via glucocorticoid blockade and immune inhibition, both in isolation and combination, provide an added means by which to confirm the model's validity. The validation of these latter predictions ultimately aims to promote translational medicine by testing potential candidate treatment targets for re-establishing healthy neuro-immune regulation.

While certainly more comprehensive than its predecessors, the neuroimmune model presented here consists of relatively coarse representations of the interplay between the cells of the brain's immune system. However, the methodology presented is general and may be used to incorporate additional elements and detail, such as the role of regulatory T cells, or the vital role of the peripheral immune system which would be an additional source of TNF-α, IL-1β, and IL-6 to further act synergistically with microglia in the brain (London et al., 2013). We expect that increased detail will lead to the emergence of additional regulatory responses rather than the elimination of the regulatory modes found here. Nonetheless, the cell types and signaling molecules represented here are known to play an important role in neuroinflammation and this simple model reveals the importance of an integrative approach to understanding chronic neuroinflammation. The findings presented here also support an alternative model for chronic illness, namely one that is not characterized by failure of individual elements, but rather with a shift in their coordinated actions away from typical healthy regulation. Finally, we emphasize that it was never our hypothesis that neuroinflammation resulted solely from the actions of homeostatic upset. Rather, we propose that homeostatic drive may be a contributor to the persistence of this condition. Natural neuron-glia interactions provide both beneficial and detrimental regulatory patterns which are resistant to change. As such, they may offer fertile ground in support of many chronic pathological processes. In turn, this may promote resistance to therapy and the natural regulatory barrier to change, even positive change, should at least be considered in the design of targeted therapeutic strategies. The knowledge of the multi-cellular regulation identified here can be used to design treatments that not only overcome, but may even harness pathological regulatory dynamics to deliver long-lasting remission (Craddock et al., 2015). Theoretically, such optimized treatment courses could eventually be discontinued once the system returns to normal regulatory behavior. This is very different from conventional long-term administration pharmacotherapy where the regulatory system is held artificially in a more desirable, but unstable state through continued intervention.

## Author contributions

TC and GB conceived and designed the computational modeling and experiments. DM and JO conceived and designed the animal modeling and experiments. TC and MR performed the computational simulation experiments. LM and KK performed the animal modeling and experiments. TC, LM, KK, and MR analyzed the data. TC, NK, MM, JO, and GB contributed reagents, materials, and analysis tools. TC, LM, KK, MR, DM, NK, MM, JO, and GB wrote the paper.

### Conflict of interest statement

The authors declare that the research was conducted in the absence of any commercial or financial relationships that could be construed as a potential conflict of interest.
