# Supplementary material for: A Logic Model of Neuronal-Glial Interaction Suggests Altered Homeostatic Regulation in the Perpetuation of Neuroinflammation
Source: Front Cell Neurosci. 2018 Oct 15;12:336. doi: 10.3389/fncel.2018.00336 (PMC6196274; doi:10.3389/fncel.2018.00336)
Supplement: Supplementary file 2 [file Table_2.DOCX]

Supplementary Material

A Logic Model of Neuronal-Glial Interaction Suggests Altered Homeostatic Regulation in the Perpetuation of Neuroinflammation

**Travis J.A. Craddock^*^, Lindsay T. Michalovicz, Kimberly A. Kelly, Mark A. Rice Jr., Diane B. Miller, Nancy G. Klimas, Mariana Morris, James P. O’Callaghan, Gordon Broderick**

*** Correspondence:** Travis J.A. Craddock: tcraddock@nova.edu

**Supplementary Table 2: Ternary OR operator**

| **A∨B** | | **B** | | |
| --- | --- | --- | --- | --- |
|  |  | **-1** | **0** | **1** |
| **A** | **-1** | -1 | 0 | 1 |
|  | **0** | 0 | 0 | 1 |
|  | **1** | 1 | 1 | 1 |
